# Hepatoprotective Effect of *Satureja Khuzestanica* Essential Oil and Vitamin E in Experimental Hyperthyroid Rats: Evidence for Role of Antioxidant Effect

**Published:** 2014-09

**Authors:** Raheleh Assaei, Fatemeh Zal, Zohreh Mostafavi-Pour, Mohammad Hossein Dabbaghmanesh, Bita Geramizadeh, Gholam Hossein Ranjbar Omrani, Naser Pajouhi

**Affiliations:** 1 Endocrine and Metabolism Research Center, Nemazee Hospital, Shiraz University of Medical Sciences, Shiraz, Iran;; 2 Razi Herbal Medicines Research Center, Lorestan University of Medical Sciences, Khorramabad, Iran;; 3 Department of Reproductive Biology, School of Advanced Medical Sciences and Technologies, Shiraz University of Medical Sciences, Shiraz, Iran;; 4 Infertility Research Center, Shiraz University of Medical Sciences, Shiraz, Iran;; 5 Recombinant Protein Lab, School of Advanced Medical Sciences and Technologies, Shiraz University of Medical Sciences, Shiraz, Iran;; 6 Maternal-Fetal Medicine Research Center, Hafez Hospital, Shiraz University of Medical Sciences, Shiraz, Iran;; 7 Department of Pathology, School of Medicine, Shiraz University of Medical Sciences, Shiraz, Iran;; 8 Department of Physiology, School of Medicine, Shiraz University of Medical Sciences, Shiraz, Iran

**Keywords:** *Satureja*, Oxidative stress, Rat

## Abstract

**Background:** Hyperthyroidism is associated with liver oxidative stress causing liver dysfunction in many hyperthyroid patients. The hepatoprotective effect of *Satureja Khuzestanica* Essential Oil (SKEO), as herbal origin antioxidant and anti-inflammatory agent on the hyperthyroidism induced hepatotoxicity and oxidative stress is investigated.

**Methods: **Adult male *sprague* dawley rats were divided into categories of; control (group C), hyperthyroid (group H), hyperthyroid with olive oil (group H+O), hyperthyroid with vitamin E (group H+E), hyperthyroid with SKEO (group H+S), combination of hyperthyroid with vitamin E and SKEO (group H+S+E). Hepatoprotective and antioxidant properties of SKEO with or without vitamin E in hyperthyroid rats were then investigated.

**Results: **Serum Aspartate Transaminase (AST) and Alanine Transaminase (ALT) activities reduced significantly in H+O, H+E, H+S and H+S+E groups in comparison with hyperthyroid rats. Enzymes activities returned to normal in H+S+E group. Hepatic Malondialdehyde (MDA) was reduced in H+E, H+S and H+S+E groups in comparison with hyperthyroid rats. The most significant MDA reduction was in the H+S+E group. Glutathione Peroxidase (GPx) and Glutathione Reductase (GR) activities increased in H+E, H+S and H+S+E groups in comparison with group H. The largest increment in GPx and GR activities were in the H+S+E group. Glutathione level did not change in any group in comparison with the control group.

**Conclusion: **Administration of SKEO has hepatoprotective effect in hyperthyroid rats and is more effective when used in combination with vitamin E.

## Introduction

Hyperthyroidism is associated with high metabolic state, oxygen consumption and reactive oxygen species (ROS) production, resulting in oxidative stress. Oxidative stress reduces the global efficacy of the antioxidant defense system,^1^^,^^2^ resulting in tissue injury by oxidative damage of biological macromulecules including lipids, proteins and DNA,^3^ causing various health issues.

Hyperthyroidism increases oxidative stress in the liver and reduces the total antioxidant power of the tissue. It causes liver dysfunction, hepatomegaly and jaundice in many hyperthyroid patients^4^ which have been ameliorated by treatment of various antioxidants.^5^^-^^8^ Nevertheless, not much attention has been paid to the application of antioxidants alone or in combination with hyperthyroidism induced oxidative stress and liver damage.

Natural products play an important role in pharmaceutical industry as well as inspiring the search for new potential sources of bioactive molecules. Very little information is available on the role of herbal origins of antioxidants in terms of being beneficial in improving hepatotoxicity and oxidative stress produced by hyperthyroidism.

*Satureja Khuzestanica Jamzad* is a medicinal plant readily found in the southern part of Iran. *Satureja Khuzestanica* Essential Oil (SKEO) has anti-inflammatory effect,^9^ ameliorates progression of diabetic nephropathy in unine phrectomized diabetic rats^10^ and improved inflammatory bowel disease by reducing oxidative stress biomarkers.^11^ SKEO also improves reproductive potential of normal^12^ and cyclophospamide treated male rats with improved body antioxidant potential.^13^ However, recent clinical trials on hyperlipidemic diabetic patients shows that administration of SKEO does not change blood oxidants levels.^14^ On the other hand carvacrol , as the most active ingredient of SKEO,^15^ has hepatoprotective effect against D-galactosamine induced hepatotoxicity in rats.^16^ Consequently, it is assumed that SKEO may have hepatoprotective effect on experimental hyperthyroid rats. Additional assessment included evaluation of antioxidant effect of SKEO in hyperthyroid rats, change in lipid peroxidation and glutathione redox status.

## Materials and Methods

*Animals*

Adult male *sprague* dawley rats weighing 200-250 g were used. Rats were cared for according to the *Guideline for the Care and Use of Laboratory Animals*. They were permitted free access to standard laboratory chow and tap water for 10-day period prior to the experimental procedure. The animals were divided into six groups each containing eight male rats. The groups were categorized as: control (group C), hyperthyroid (group H), hyperthyroid with olive oil (group H+O), hyperthyroid with vitamin E supplied by sigma chemical CO (200 mg/kg body weight) ( group H+E), hyperthyroid with SKEO (225mg/kg body weight) (group H+S), hyperthyroid with vitamin E (200 mg/kg body weight) in combination with SKEO (225mg/kg body weight) (group H+S+E). Vitamin E and SKEO dosages were selected based on past pilot studies and experience. These were diluted with olive oil with a final volume of 1 ml and all animals were gavage daily by the solution for 30-day period.^8^^,^^11^ Hyperthyroidism in rats was achieved by administering 0.0012% L-thyroxin, (Iran Hormone CO, Iran) in drinking water for the same period.^8^ On the last day, rats were sacrificed by decapitation, blood samples were collected immediately and serum samples were stored at -20°C. Their livers were removed, cleaned by phosphate buffer saline 0.1 M (pH 7.4) at 4°C and immediately processed for biochemical estimation. Serum TSH, total T3 and T4 concentrations, ALT and AST activities were assayed. In liver homogenates MDA, GSH levels and GPx, GR activities were analyzed. For histological study, specimens were secured in 10% neutral formalin.

*Chemicals*

Thiobarbituric acid, glutathione reductase, tert-butyl hydroperoxide (t-BuOOH) and 1, 1, 3, 3,-tetraethoxy propane (TEP) were purchased from Sigma (St Louis, MO, USA). Na2-NADPH was obtained from Fluka (Buchs, Switzerland). Hydrogen peroxide (H2O2) and sodium azide were obtained from Merck (Darmstadt, Germany). All other analytically pure reagents were obtained from other commercial sources. 

*Determination of T3, T4 and TSH*

Rat TSH ELISA kit (Cusabio Biotech Co., LTD), total T3 and T4, RIA kits (Bechman coulter Co, Check) were used to determine TSH and total T3 and T4 serum concentration.

*Determination of Liver Enzymes*

Activities of aspartate transaminase (AST) and alanine transaminase (ALT) in the sera were measured by colorimetric method (A25-Autoanalyser, Spain) as indices of hepatic injury. Kits were obtained from Biosystem Co. (Spain). Results were expressed as units of AST and ALT activity/L serum.

*Preparation of the Essential Oil*

The aerial parts of the plant were collected during flowering season from Khoramabad, the province of Lorestan, Iran. The plant was identified by the Department of Botany of the Forests and Rangeland Research Institute (TARI) in Tehran. A voucher specimen (No.58416) was deposited at TARI herbarium. Plant samples were air-dried at ambient temperature in shade and hydrodistillation in a clevenger-type apparatus for 5 h, resulting in yellow oil with 0.9% yield. The oil was dried over anhydrous sodium sulfate and stored at 4°C. The density of the essence was 0.98.

*Preparation of Liver Extract*

The frozen liver tissue samples were cut into small pieces and homogenized in ice-cold saline to produce 10% (w/v) homogenates. These were centrifuged at 10,000 g for 1 h at 4°C with bench top centrifuge (Carlbcam, Germany). Supernatants were used for the measurement of Thiobarbituric acid-reactive-substances (TBARS), GSH levels, GR and GPx activities. The protein in the liver supernatants was measured by the Bradford method, using bovine serum albumin as a standard.^17^

*Determination of TBARS Concentration*

TBARS assay values are typically reported in malondialdehyde (MDA) equivalents. Therefore, TBARS assay was used to estimate peroxidation of lipids in membrane and biological systems. The liver TBARS was assayed by a colorimetric method as described by Zal et al.^18^ The TBARS concentration was calculated using 1, 1, 3, 3-tetraethoxy propane (TEP) as standard. The results were expressed as nmol/mg protein of the liver supernatant.

*Glutathione Peroxidase (GPx) Assay*

The glutathione peroxidase (GPx) activity of samples was measured by continuous monitoring of the regeneration of the reduced glutathione (GSH) from oxidized glutathione GSSG upon the action of GR and NADPH according to the method of Fecondo and Augusteyn.^19^ To the 750 µL reaction mixture containing 0.3 mmol/L EDTA, 0.1 mmol/L NADPH, 0.5 units GR and 0.5 mmol/L Na_2_N_3_ in 50 mmol/L phosphate buffer (pH 7.2), 50 µL plasma and 100 µL of 2.5 mmol/L GSH were added. Tubes in which distilled water was substituted for GSH were included as controls. Following the addition of 100 µL of 0.4 mmol/L tert-butyl hydroperoxide (t-BuOOH) to each experimental and control tubes, the decrease in NADPH absorbance at 340 nm was measured at 37°C for 3 min. The decrease in absorbance, reflecting the oxidation of NADPH which is directly proportional to the GPx activity in the sample, was followed at 340 nm. Results were expressed as units of GPx activity/gram of protein.

*Glutathione Reductase Assay*

The enzyme, GR, catalyzes the reduction of GSSG to GSH and is essential for glutathione redox cycle in order to maintain adequate levels of reduced cellular GSH. During the reduction of GSSG by GR, one molecule of NADPH is consumed for the reduction of each molecule of reduced GSSG. Therefore, the reduction of GSSG by GR can be determined by measuring the consumption of NADPH. The activity of GR was assayed using the method described by Carlberg and Mannervik^20^ with minor modifications. The GR assay was performed in a cuvette containing 1M Tris-HCl buffer +5 mM EDTA (pH 8.0), 0.033M GSSG, 2mM NADPH, and a sample in final volume of 1.0 mL. Decrease in the absorbance, which reflects the oxidation of NADPH during reduction of GSSG by GR present in the sample, was monitored spectrophotometrically at 340 nm. Results were expressed as units of GR activity/gram of protein.^21^

*Determination of GSH in Liver Homogenates*

The assay of glutathione (GSH) with DTNB was performed, followed by the standard Ellman’s method^18^ for liver homogenates. 0.5ml (0.001 M) of 5, 5-dithiobis, 2-nitrobenzoic acid (DTNB) in a phosphate buffer was added to all homogenates. The absorbance of the resulting product was observed after 5 minutes at 412 nm using UV/Visible double beam spectrophotometer. GSH level was then determined from the standard curve of reduced GSH obtained with 0.2, 0.4, 0.6, 0.8 and 1 mM GSH concentration. Total GSH content was expressed as nmol GSH/mg protein.

*Histological Study*

Liver tissue specimens from all experimental groups were taken for light microscopy. The specimens were fixed in 10% neutral formalin, dehydrated in alcohol and embedded in paraffin. Sections 5 μm-thick were stained with Hematoxylen-Eosine and examined by Olympus BH2.

*Statistical Analysis*

All data were presented as mean±standard error and were analyzed by Kolmogrov-Smirnov test. In this test, all data had P>0.05 and thus were considered as parametric data. Consequently, they were subjected to one way ANOVA followed by Tukey’s test. Minimal statistical significance was accepted as P<0.05.

## Results

*Serum T3, T4 and TSH Level*

A significant increase in serum T3 and T4 concentrations was observed in all thyroxin treated rats. TSH level significantly reduced in all T4 administered animals compared to that in the control group (data not shown). 

*Liver Lipid Peroxidation*

Hepatic MDA increased significantly in group H compared to that in the control group. On the other hand, MDA was decreased in groups H+O, H+E, H+S and H+S+E. The most significant decline was observed in H+S+E group and minimal decrease in H+O group. There were no significant differences between H+E and H+S groups (table1).

**Table1:** GPx (U/gram of protein), GR (U/gram of protein) activities and MDA (nmol/mg protein), GSH (nmol/mg protein) content in rat liver homogenates in different groups after 30 days treatment

**Groups**	**MDA**	**GPx**	**GR**	**GSH**
Control (C)	1.72±0.14^a^	86.72±2.98^ a^	64.27±1.7^ a^	0.21±0.015^ a^
T4 (H)	4.38±1.42^b^	54.22±2.57 ^b^	49.58±3.24^ b^	0.21±0.026^a^
T4+olive oil (H+O)	2.7±0.32^c^	67.37±3.79^ c^	47.15±2.6^b^	0.18±0.004^ a^
T4+vitamin E, 200 mg/kg (H+E)	1.52±0.28^a^	82.89±5.69^a^	65±4.03^a^	0.19±0.012^ a^
T4+SKEO, 225 mg/kg (H+S)	1.47±0.34^ a^	80.13±4.44^ a^	69.9±2.11^ a^	0.18±0.015^ a^
T4+ SKEO, 225 mg/kg+vitamin E, 200 mg/kg (H+S+E)	1.01±0.16^d^	96.52±5.8^d^	77.5±4.06^d^	0.2±0.012^ a^

Data are mean±SEM (n=8). Data having different superscript letters (a-d) are significant at P<0.05. Data having the same letter have not significant differences.

*Liver Glutathione Peroxidase and Glutathione Reductase Activities and Glutathione Content*

GPx and GR activities decreased following T4 administration. GPx and GR activities significantly increased in H+E, H+S and H+S+E groups. Higher increase in GPx and GR activities was observed when a combination of SKEO and vitamin E were used. GSH level did not change in any group compared with the control group (table 1).

*Serum Transaminase Activity*

The activities of ALT and AST were elevated significantly in hyperthyroid rats compared with the control group. ALT and AST activities reduced significantly in all test groups compared to that in the hyperthyroid rats (table 2).The enzymes activities returned to normal in H+S+E group.

**Table2:** Serum ALT (U/L) and AST (U/L) activities in different groups after 30 days treatment

**Groups**	**ALT**	**AST**
Control (C)	68±5.23^a^	180_±_6.8^a^
T4 (H)	203.83±26.42^b^	314.33±37.88^b^
T4+olive oil (H+O)	163.00±14.66^c^	244.6±16.66^c^
T4+vitamin E, 200 mg/kg (H+E)	138.40±12.71^d^	203±19.73^d^
T4+SKEO, 225 mg/kg (H+S)	116.00±16.5^d^	174±28.18^a^
T4+SKEO, 225 mg/kg+vitamin E, 200 mg/kg (H+S+E)	96.86±15.09^a^	166±10.9^a^

Data are mean±SEM (n=8). Data having different superscript letters (a-d) are significant at P<0.05. Data having the same letter have not significant differences.

*Histological Study*

**Figure 1:**
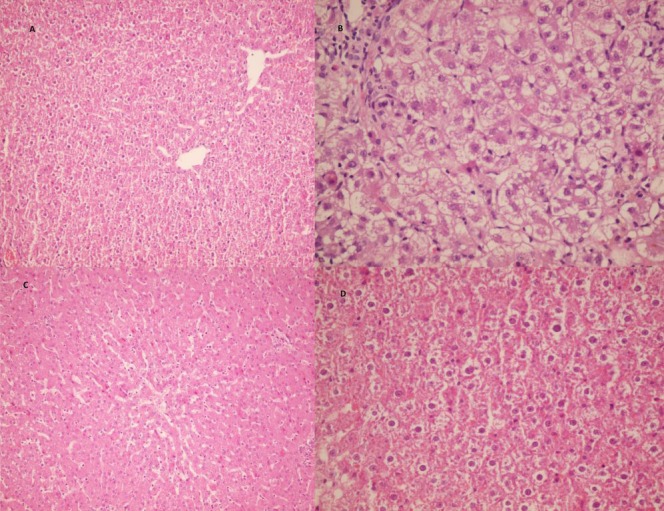
Representative histopathological profiles on the liver of intact control (a), hyperthyroid (b), hyperthyroid with *Satureja khuzestanica* essential oil (225mg/kg) treated rats(c), hyperthyroid with olive oil treated rats (d).

Liver tissue of the control group showed normal structure under light microscopic examination (figure 1A), but the liver tissue of hyperthyroid animals had cytoplasmic vacuolization, focal necrosis, fragmented cytoplasm, apoptotic hepatocytes and inflammatory cell infiltration. There were no steatosis and cholestasis in this group (figure 1B). In groups H+E, H+S and H+S+E, histoarchitecture of the liver was near normal (figure 1C). In H+O group, there was less cytoplasmic vacuolization and focal fragmented cytoplasm, no focal necrosis was observed (figure 1D).

## Discussion

In this investigation, a hyperthyroidism model was required and as observed in all L-thyroxin treated rats, serum T3 and T4 elevation and TSH level reduction confirmed hyperthyroid induced by L-thyroxin. 

Hepatic toxicity as a well-known adverse effect of hyperthyroidism is characterized by a rise in serum ALT and AST activities as liver damage biomarker enzyme. Diagnosis of liver disease and the extent of damage to the structural integrity of the liver are commonly evaluated by serum AST and ALT activities.^22^ Results from this study indicates that hyperthyroidism induced marked hepatic toxicity through induction of oxidative toxic stress that is prevented by SKEO and vitamin E.

SKEO seems to preserve the structural integrity of hepatocellular membrane as evidenced from significant reduction in the activities of the liver enzymes. It should be noted that there is no evidence on hepatoprotective effect of SKEO yet. However, major monoterpenic phenol of SKEO (i.e. carvacrol) has antioxidant,^23^ anti- inflammatory^24^ and hepatoprotective potential.^16^ Therefore, SKEO may preserve structural integrity of hepatocellular membrane through carvacrol which was reported previously in D-galactosamine-induced hepatotoxicity in rats.^16^

Moreover, a substantial increase in lipid peroxidation in the hyperthyroid rats as assessed by liver MDA values was observed. This finding indicate that hyperthyroidism increases the production of active oxygen species, generation of hydroxyl radicals, which readily start the free radical mediated lipid peroxidation and results in increased MDA production. This effect can be explained through hormone induced calcium efflux in cytosol and imbalance of calcium homeostasis^25^ which in turn activates arachidonic acid cascade that produces ROS.^26^

It seems that the above cascade of reactions was controlled differently by vitamin E and SKEO. Vitamin E is a potent lipid soluble antioxidant in biological systems with the ability to directly quench free radicals and also check lipid peroxidation by limiting the propagation change of lipid peroxidation.^16^ On the other hand SKEO, an antioxidant and anti-inflammatory agent may inhibit COX2 enzyme through carvacrol,^27^ causing inhibition of arachidonic acid metabolism and ROS production that might reduce lipid peroxidation production.

GR is the major enzyme for glutathione reduction and replenishes the GSH pool by oxidizing NADPH, produced in pentose phosphate pathway, while GPx is a GSH utilizing enzyme. GPx is an antioxidant enzyme which catalyses H_2_O_2_ and lipid hydroperoxides reduction, with oxidation of GSH into GSSG. Therefore, GPx prevents the generation of hydroxyl radical. It is found that L-thyroxin administration impaired the rat liver GPx and GR activities. Hyperthyroidism increases the production of H_2_O_2_ in the liver mitochondria^28^ which easily diffuses into the cytosol. The other important source of H_2_O_2_ in cytosol is proxisomal B-oxidation of fatty acid, a process stimulated by thyroid hormones.^29^ Therefore, decreased GPx and GR activities in hyperthyroid rats can be due to the damage to enzymes by lipid peroxidation products which are strongly induced in hyperthyroid rats. These products cause a loss of GPx and GR activities, probably by modification of selenocycteine residue at the active site of the enzyme.^30^^,^^31^ Lower activities of these enzymes will result in accumulation of highly reactive free radicals, leading to deleterious effects such as loss of cell membrane integrity and membrane function.^32^ SKEO supplementation with vitamin E significantly increased the activities of GPx and GR compared to that in SKEO or vitamin E administration alone.^33^ In accordance with the effect of carvacrol in scavenging superoxide radicals and hydrogen peroxide and reduction of the free-radical-mediated inactivation of enzyme proteins , SKEO administration may decrease the workload of the enzymatic antioxidants, GPx and GR, and thereby maintaining their activities through carvacrol as a powerful antioxidant substance. This effect was significantly more when SKEO and vitamin E, as powerful antioxidant, were administered.

It is shown that cellular level of GSH did not change in any group compared to that in the control group. This might be due to the reduced activities of both GPx and GR in hyperthyroid liver, preventing optimum GSH utilization and recycling. There is high controversy on the GSH and GSSG pools, including depletion,^34^ increase^35^ and unaltered^36^ GSH and GSSG pools in experimental hyperthyroid rat liver. Such debate may be the result of age variation of the experimental animals, dose and duration of L-thyroxin induced hyperthyroidism that influences glutathione content in the experimental hyperthyroid rat liver.^37^

In histological study, SKEO and vitamin E supplement normalized inflammation, necrosis and apoptosis in liver tissue of hyperthyroid rats. Fernandez et al. showed that hyperthyroid state in rat increases circulating levels of TNF-α by actions exerted at the Kupffer cell level. These are related to the oxidative stress status established in the liver by thyroid calorigenesis. This inflammatory cytokine is involved in the pathogenesis of hyperthyroid induced liver injury. It has become evident that TNFα triggers apoptosis and/or necrosis of the hepatocytes in vivo.^38^ Recently, Guimaraes et al.^24^ reported that carvacrol inhibits the development of edema induced by carrageenan, and also significantly decreases TNFα levels. Therefore, SKEO improved hepatocyte necrosis and apoptosis induced by hyperthyroidism probably through decreasing TNFα level in the liver. Also, carvacrol is a potential medicine for the treatment of inflammatory diseases through inhibition of histamine release and leukocyte migration.^39^ Therefore, the innate anti-inflammatory property of the essential oil may explain the observed anti-inflammatory effect in the liver. SKEO is more effective when used with vitamin E. Therefore, it is strongly recommended to ascertain hepatoprotective effect of SKEO with vitamin E in hyperthyroid patients as a clinical trial study. Evaluation of more mechanism(s) is required.

## Conclusion

SKEO has hepatoprotective effect in hyperthyroid rats possibly through improvement of oxidative stress and its anti-inflammatory effects. Its effect is intensified when used in combination with vitamin E. 

## References

[1] Ourique GM, Finamor IA, Saccol EM, Riffel AP, Pês TS, Gutierrez K (2013). Resveratrol improves sperm motility, prevents lipid peroxidation and enhances antioxidant defences in the testes of hyperthyroid rats. Reprod Toxicol.

[2] Messarah M, Saoudi M, Boumendjel A, Boulakoud MS, Feki AE (2011). Oxidative stress induced by thyroid dysfunction in rat erythrocytes and heart. Environ Toxicol Pharmacol.

[3] Messarah M, Boumendjel A, Chouabia A, Klibet F, Abdennour C, Boulakoud MS (2010). Influence of thyroid dysfunction on liver lipid peroxidation and antioxidant status in experimental rats. Exp Toxicol Pathol.

[4] Khemichian S, Fong TL (2011). Hepatic dysfunction in hyperthyroidism. Gastroenterol Hepatol (N Y).

[5] Giriş M, Erbil Y, Depboylu B, Mete O, Türkoğlu U, Abbasoğlu SD (2010). Heme oxygenase-1 prevents hyperthyroidism induced hepatic damage via an antioxidant and antiapoptotic pathway. J Surg Res.

[6] Makay O, Yenisey C, Icoz G, Genc Simsek N, Ozgen G, Akyildiz M (2009). The role of allopurinol on oxidative stress in experimental hyperthyroidism. J Endocrinol Invest.

[7] Panda S, Kar A (2007). Amelioration of L-thyroxine-induced hyperthyroidism by coumarin (1,2-benzopyrone) in female rats. Clin Exp Pharmacol Physiol.

[8] Subudhi U, Das K, Paital B, Bhanja S, Chainy GB (2008). Alleviation of enhanced oxidative stress and oxygen consumption of L-thyroxine induced hyperthyroid rat liver mitochondria by vitamin E and curcumin. Chem Biol Interact.

[9] Amanlou M, Dadkhah F, Salehnia A, Farsam H, Dehpour AR (2005). An anti-inflammatory and anti-nociceptive effects of hydroalcoholic extract of Satureja khuzistanica Jamzad extract. J Pharm Pharm Sci.

[10] Tavafi M, Ahmadvand H, Tamjidipoor A, Delfan B, Khalatbari AR (2011). Satureja khozestanica essential oil ameliorates progression of diabetic nephropathy in uninephrectomized diabetic rats. Tissue Cell.

[11] Ghazanfari G, Minaie B, Yasa N, Nakhai LA, Mohammadirad A, Nikfar S (2006). Biochemical and histopathological evidences for beneficial effects of satureja khuzestanica jamzad essential oil on the mouse model of inflammatory bowel diseases. Toxicol Mech Methods.

[12] Haeri S, Minaie B, Amin G, Nikfar S, Khorasani R, Esmaily H (2006). Effect of Satureja khuzestanica essential oil on male rat fertility. Fitoterapia.

[13] Rezvanfar M, Sadrkhanlou R, Ahmadi A, Shojaei-Sadee H, Rezvanfar M, Mohammadirad A (2008). Protection of cyclophosphamide-induced toxicity in reproductive tract histology, sperm characteristics, and DNA damage by an herbal source; evidence for role of free-radical toxic stress. Hum Exp Toxicol.

[14] Vosough-Ghanbari S, Rahimi R, Kharabaf S, Zeinali S, Mohammadirad A, Amini S (2010). Effects of Satureja khuzestanica on Serum Glucose, Lipids and Markers of Oxidative Stress in Patients with Type 2 Diabetes Mellitus: A Double-Blind Randomized Controlled Trial. Evid Based Complement Alternat Med.

[15] Hashemi MB, Niakousari M, Saharkhiz MJ, Eskandari MH (2012). Effect of Satureja khuzestanica essential oil on oxidative stability of sunflower oil during accelerated storage. Nat Prod Res.

[16] Aristatile B, Al-Numair KS, Veeramani C, Pugalendi KV (2009). Effect of carvacrol on hepatic marker enzymes and antioxidant status in D-galactosamine-induced hepatotoxicity in rats. Fundam Clin Pharmacol.

[17] Bradford MM (1976). A rapid and sensitive method for the quantitation of microgram quantities of protein utilizing the principle of protein-dye binding. Anal Biochem.

[18] Zal F, Mostafavi-Pour Z, Vessal M (2007). Comparison of the effects of vitamin E and/or quercetin in attenuating chronic cyclosporine A-induced nephrotoxicity in male rats. Clin Exp Pharmacol Physiol.

[19] Fecondo JV, Augusteyn RC (1983). Superoxide dismutase, catalase and glutathione peroxidase in the human cataractous lens. Exp Eye Res.

[20] Carlberg I, Mannervik B (1985). Glutathione reductase. Methods Enzymol.

[21] Mostafavi-Pour Z, Zal F, Monabati A, Vessal M (2008). Protective effects of a combination of quercetin and vitamin E against cyclosporine A-induced oxidative stress and hepatotoxicity in rats. Hepatol Res.

[22] Amin A, Hamza AA (2005). Oxidative stress mediates drug-induced hepatotoxicity in rats: a possible role of DNA fragmentation. Toxicology.

[23] Ozkan A, Erdogan A (2012). A comparative study of the antioxidant/prooxidant effects of carvacrol and thymol at various concentrations on membrane and DNA of parental and drug resistant H1299 cells. Nat Prod Commun.

[24] Guimarães AG, Xavier MA, de Santana MT, Camargo EA, Santos CA, Brito FA (2012). Carvacrol attenuates mechanical hypernociception and inflammatory response. Naunyn Schmiedebergs Arch Pharmacol.

[25] Del Viscovo A, Secondo A, Esposito A, Goglia F, Moreno M, Canzoniero LM (2012). Intracellular and plasma membrane-initiated pathways involved in the [Ca2+]i elevations induced by iodothyronines (T3 and T2) in pituitary GH3 cells. Am J Physiol Endocrinol Metab.

[26] Czapski GA, Czubowicz K, Strosznajder RP (2012). Evaluation of the antioxidative properties of lipoxygenase inhibitors. Pharmacol Rep.

[27] Landa P, Kokoska L, Pribylova M, Vanek T, Marsik P (2009). In vitro anti-inflammatory activity of carvacrol: Inhibitory effect on COX-2 catalyzed prostaglandin E(2) biosynthesis. Arch Pharm Res.

[28] Venditti P, De Rosa R, Di Meo S (2003). Effect of thyroid state on H2O2 production by rat liver mitochondria. Mol Cell Endocrinol.

[29] Weitzel JM, Radtke C, Seitz HJ (2001). Two thyroid hormone-mediated gene expression patterns in vivo identified by cDNA expression arrays in rat. Nucleic Acids Res.

[30] Dabbaghmanesh MH, Sadegholvaad A, Ejtehadi F, Omrani G (2007). Low serum selenium concentration as a possible factor for persistent goiter in Iranian school children. Biofactors.

[31] Miyamoto Y, Koh YH, Park YS, Fujiwara N, Sakiyama H, Misonou Y (2003). Oxidative stress caused by inactivation of glutathione peroxidase and adaptive responses. Biol Chem.

[32] Rolo AP, Teodoro JS, Palmeira CM (2012). Role of oxidative stress in the pathogenesis of nonalcoholic steatohepatitis. Free Radic Biol Med.

[33] Beena, Kumar D, Rawat DS (2013). Synthesis and antioxidant activity of thymol and carvacrol based Schiff bases. Bioorg Med Chem Lett.

[34] Chattopadhyay S, Sahoo DK, Subudhi U, Chainy GB (2007). Differential expression profiles of antioxidant enzymes and glutathione redox status in hyperthyroid rats: a temporal analysis. Comp Biochem Physiol C Toxicol Pharmacol.

[35] Mogulkoc R, Baltaci AK, Oztekin E, Sivrikaya A, Aydin L (2006). Effects of hyperthyroidism induced by L-thyroxin administration on lipid peroxidation in various rat tissues. Acta Biol Hung.

[36] Das K, Chainy GB (2001). Modulation of rat liver mitochondrial antioxidant defence system by thyroid hormone. Biochim Biophys Acta.

[37] Saicić ZS, Mijalković DN, Nikolić AL, Blagojević DP, Spasić MB (2006). Effect of thyroxine on antioxidant defense system in the liver of different aged rats. Physiol Res.

[38] Fernandez V, Videla LA, Tapia G, Israel Y (2002). Increases in tumor necrosis factor-alpha in response to thyroid hormone-induced liver oxidative stress in the rat. Free Radic Res.

[39] Fachini-Queiroz FC, Kummer R, Estevão-Silva CF, Carvalho MD, Cunha JM, Grespan R (2012). Effects of Thymol and Carvacrol, Constituents of Thymus vulgaris L. Essential Oil, on the Inflammatory Response. Evid Based Complement Alternat Med.

